# Adenocarcinoma arising from a heterotopic pancreas in the first portion of the duodenum: a case report

**DOI:** 10.1186/s40792-020-00903-z

**Published:** 2020-06-18

**Authors:** Teruya Minami, Takuro Terada, Takeshi Mitsui, Yasuni Nakanuma

**Affiliations:** 1 Department of Surgery, Fukui-ken Saiseikai Hospital, Fukui, Japan; 2 Department of Pathology, Fukui-ken Saiseikai Hospital, Fukui, Japan

**Keywords:** Heterotopic pancreas, Adenocarcinoma, SSPPD

## Abstract

**Background:**

Heterotopic pancreas (HP) is defined as pancreatic tissue in organs with no anatomical continuity with the orthotopic pancreas. Based on the number of cases reported in the literature between the year 2000 and 2020, HP is rarely found causing malignant transformation of the duodenum. We herein report a case of adenocarcinoma arising from the HP in the first portion of the duodenum.

**Case presentation:**

A 77-year-old Japanese man presented to our hospital with epigastric pain. Despite having undergone laparoscopic surgery for early sigmoid colon cancer a month earlier, serum levels of tumor-specific antigens, such as CA19-9, were elevated. After undergoing a series of radiologic examinations, the first portion of the duodenum was found thickened. However, a biopsy of the lesion showed no malignancy. Four months later, follow-up computed tomography (CT) scans showed that the lesion was thicker and involved the gastroduodenal artery (GDA), suggesting tumor invasion. A new biopsy did not detect the malignancy. However, serum tumor-specific antigen levels increased, especially duke pancreatic monoclonal antigen type 2 (5287 U/mL), in the absence of tumor in the orthotopic pancreas. The follow-up CT imaging showed a malignant tumor in the first portion of the duodenum. Five months later, we performed a subtotal stomach-preserving pancreaticoduodenectomy (SSPPD) for duodenal or HP cancer in the first portion of the duodenum, finding a lesion from the pyloric bulbs to the first portion of the duodenum, which invaded the adjacent pancreas and GDA. The pathological examination of the specimens revealed adenocarcinoma arising from HP. Nine months after surgery, no recurrence was found by radiologic imaging or tumor-specific antigen laboratory testing.

**Conclusions:**

HP adenocarcinoma is rare and difficult to diagnose preoperatively due to its submucosal location. Therefore, a careful follow-up with blood testing and radiologic imaging, as well as diagnostic surgery, is recommended.

## Background

Heterotopic pancreas (HP), also called ectopic or aberrant pancreas, is defined as pancreatic tissue located in organs with no anatomical continuity with the orthotopic pancreas. HP commonly occurs at the submucosal layer of the gastrointestinal tract and is difficult to diagnose preoperatively. On the basis of abdominal surgery evidence, the incidence of HP is estimated to range from 0.25 to 1.2% [1, 2]. Notably, the incidence of malignant transformation in HP is extremely rare. To our knowledge, only 12 cases of HP cancer of the duodenum are found in the literature since the year 2000 after performing a PubMed search (keywords: heterotopic OR ectopic OR aberrant pancreas, carcinoma). Herein, we describe a case of adenocarcinoma arising from HP in the first portion of the duodenum and include a review of the literature.

## Case presentation

**Fig. 1:**
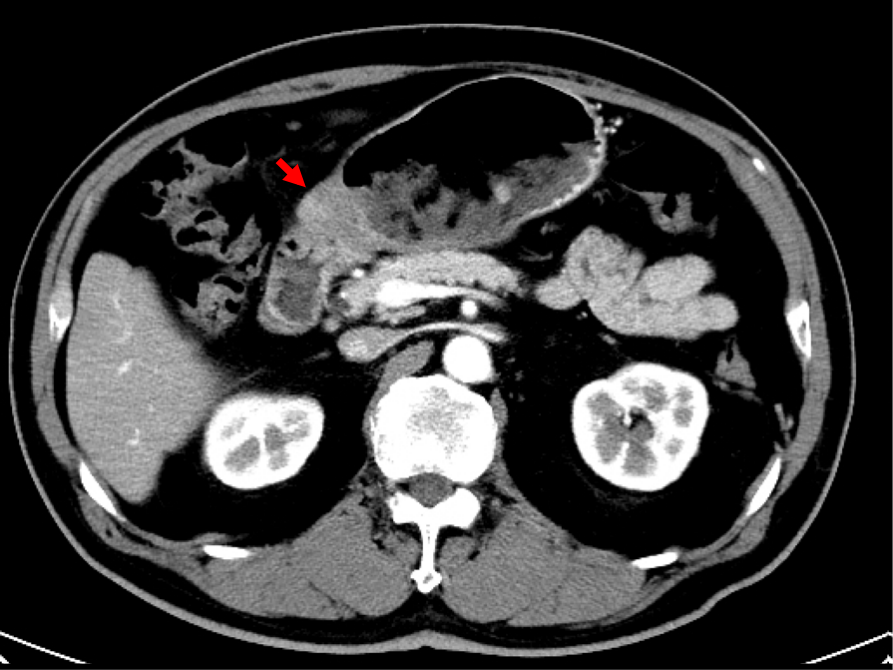
Abdominal CT scan showing a thickened wall in the first portion of the duodenum (arrow)

**Fig. 2:**
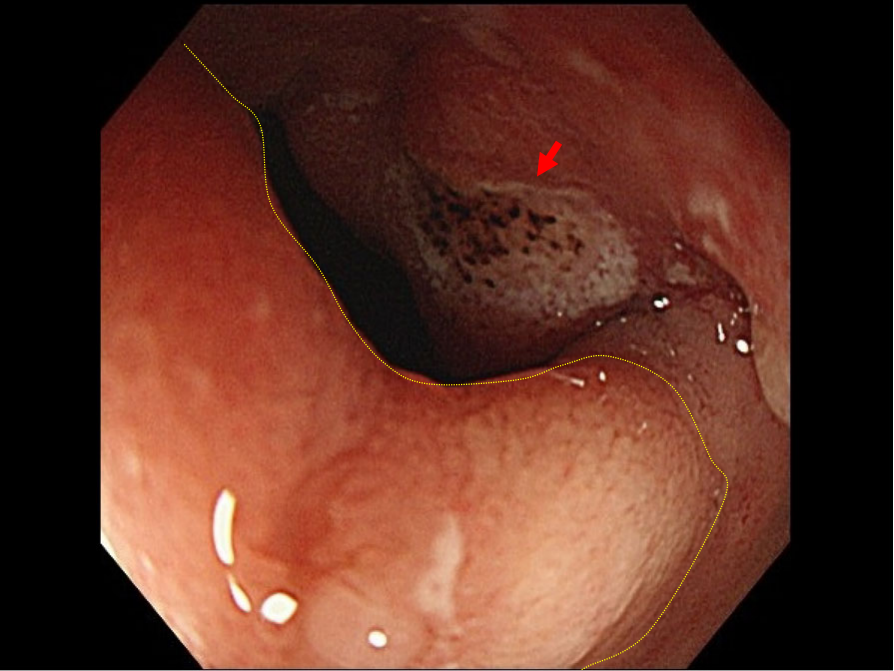
Gastroscopic image showing a slight pyloric stenosis with a small ulcer (arrow). The lesion was covered with normal mucosa (yellow dotted circles)

**Fig. 3:**
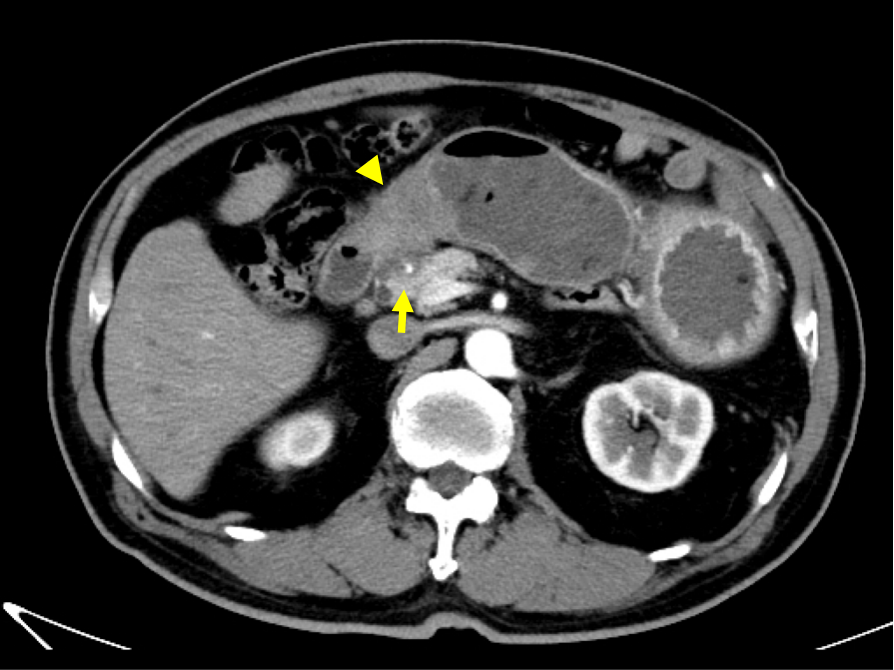
Four months later, abdominal CT scan showing a more thickened wall than that observed in previous CT scans (arrow heads). The lesion involved the surrounding tissue of the gastroduodenal artery, suggesting invasion (arrow)

**Fig. 4:**
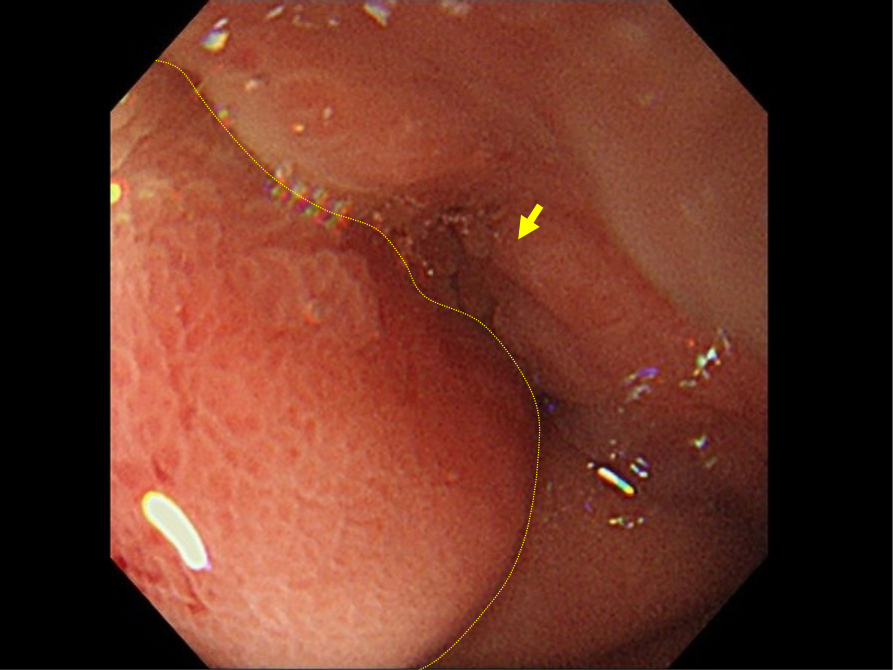
Four months later, gastroscopic image showing a pyloric stenosis worse than that observed in the previous endoscopy. Normal mucosa of the lesion did not change (yellow dotted circles)

A 77-year-old Japanese man presented to our hospital with epigastric pain and a medical history of type 2 diabetes and duodenal ulcer. One month earlier, the patient had undergone laparoscopic surgery for early stage sigmoid colon cancer, classified as pathological T1N0M0 stage I according to the 7th edition of the Union for International Cancer Control (UICC)’s TMN classification of malignant tumors. The results of the physical examination were unremarkable and his vital signs were within the reference range. Laboratory test results showed elevated serum levels of tumor-specific antigen: carbohydrate antigen 19­9 (CA19-9), 1130 U/mL (reference range, 0–37 U/mL). Enhanced multi-detector computed tomography images showed a significant gastric distension and slight thickening of the first portion of the duodenum (Fig. 1). Esophagogastroduodenoscopy results revealed an ulcer scar with slight stenosis and no evidence of malignancy after biopsy (Fig. 2). The patient was followed up for a short term on an outpatient basis. We evaluated the thickening lesion by laboratory testing, radiologic imaging, and endoscopic examination every 4 months. Four months later, his subjective symptoms remained unchanged, but follow-up CT showed thickening of the stenosis-surrounding tissue with the involvement of the gastroduodenal artery (GDA), suggesting tumor invasion (Fig. 3). Endoscopy examination was challenging due to the difficultly in passing through the thickening lesion (Fig. 4). CA19-9 serum levels remained elevated, and serum level of duke pancreatic monoclonal antigen type 2 (DUPAN-2), which is a pancreatic tumor-specific antigen, was especially elevated, 5287 U/mL (reference range, 0–37 U/mL). Because CT images showed no evidence of the tumor in the orthotopic pancreas, we suspected that the malignancy could be located in heterotopic pancreas tissue. After 5 months of diagnostic intervention, we performed a subtotal stomach-preserving pancreatoduodenectomy in the first portion of the duodenum to treat the duodenal and presumed heterotopic pancreatic cancer.

**Fig. 5:**
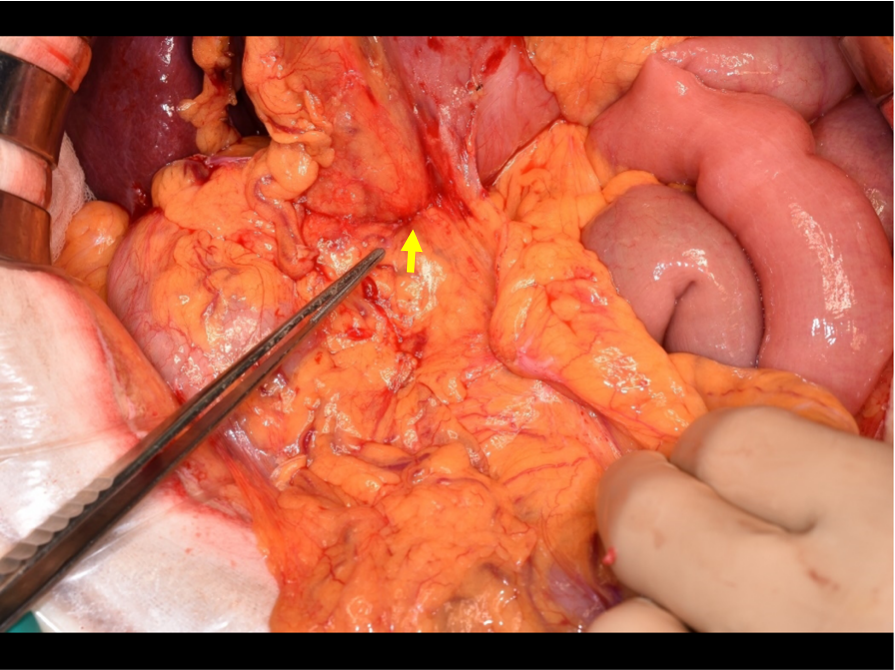
Gross image of the intraoperative finding of an elastic hard tumor at the duodenum bulbs invading the adjacent pancreas (arrow)

Intraoperative findings included the identification of an elastic hard tumor at the duodenal bulbs invading the adjacent pancreas and GDA (Fig. 5). The gastrointestinal reconstruction strategy involved the modified Child method with Kakita’s technique for pancreatojejunostomy.

**Fig. 6:**
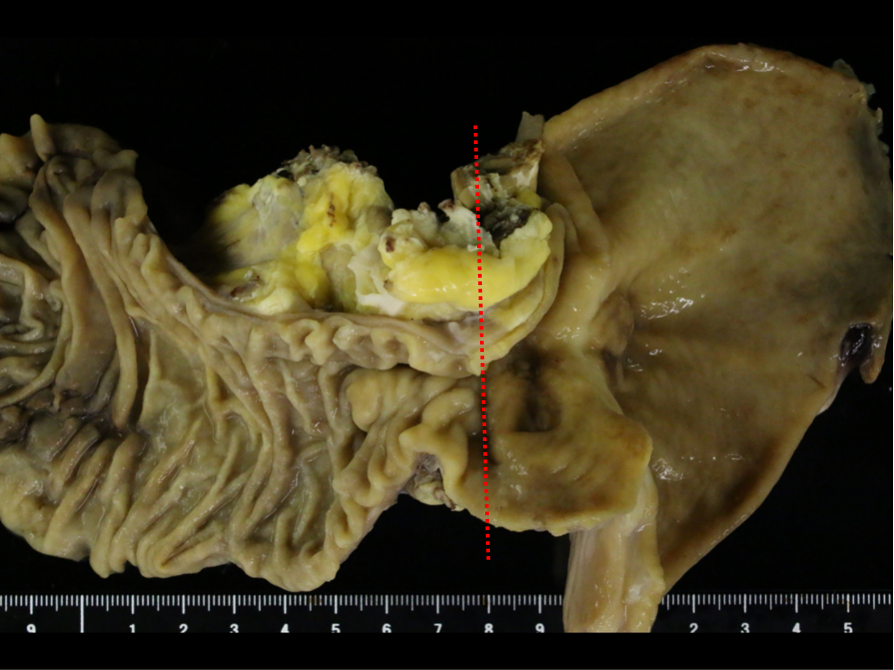
Photograph of the resected specimen revealing stenosis from the pyloric bulb to the first portion of the duodenum with normal mucosal tissue (red dotted line: Fig. 7a cutting line)

**Fig. 7:**
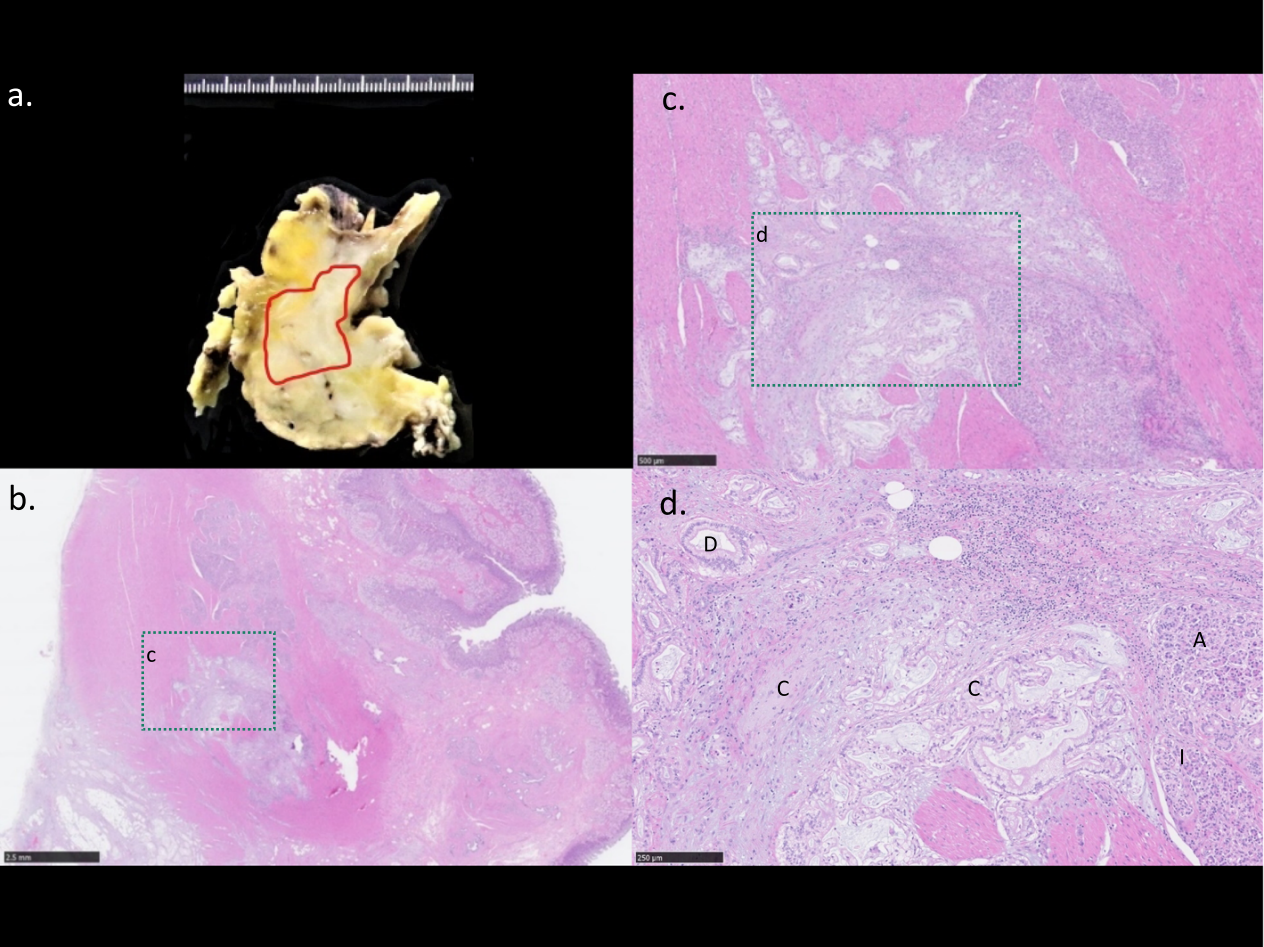
Gross and histopathological findings: **a** cut surface of the tumor showing a yellowish-white mass (red line circles) in the first duodenum wall. **b** Heterotopic pancreas (HP) with moderately differentiated adenocarcinoma tissue expanded from the submucosal layer to the muscularis propria of the first portion of the duodenum. Hematoxylin and eosin staining (× 4). **c** × 5 Magnification of **b**. **d** HP tissue consisting of acini (A), ducts (D), Langerhans islet cells (I), and adenocarcinoma cells arising from the HP (C). Hematoxylin and eosin staining (× 40)

The resected specimen had severe stenosis from the pyloric bulb to the first portion of the duodenum, whereas the mucosa appeared normal (Fig. 6). The tumor was white-yellow in color on its cut surface and had a diameter of 55 mm. A moderately differentiated tubular adenocarcinoma continuous with the heterotopic pancreatic tissue expanded from the submucosal layer to the muscularis propria of the first portion of the duodenum (Fig. 7a–c). The pancreatic tissue consisted of acini, ducts, and Langerhans islet cells (Fig. 7d) and was classified as Heinrich’s type I HP. Finally, pathological diagnosis was an adenocarcinoma arising from the HP in the first portion of the duodenum.

The postoperative course was uneventful, and the patient was discharged 25 days after surgery. One month after surgery, serum carcinoembryonic antigen level was 2.9 U/mL; CA19-9, 1.133 U/mL; and DUPAN-2, 1.613 U/mL. The patient underwent 5 courses of oral tegafur-gimeracil-dihydropyrimidine dehydrogenase (TS-1) as adjuvant chemotherapy and has remained free of disease 9 months after surgery, with no recurrence found by radiologic imaging.

## Discussion

Heterotopic pancreas (HP), also called ectopic or aberrant pancreas, is defined as pancreatic tissue in organs with no anatomical continuity with the orthotopic pancreas. Shultz first reported the existence of HP in 1727. Almost 200 years later, in 1909, Von Heinrich classified HP tissue into four histopathologic types, by the presence or absence of pancreatic ducts, acini, and islet cells [3, 4]. Commonly, HP is a benign disease comprising submucosal tumors (SMT) in the gastrointestinal tract, in organs such as the stomach, duodenum, jejunum, mesocolon, and Meckel’s diverticulum. Thus, HP initially identified as SMT by radiologic or endoscopic imaging is rarely biopsied for a definitive diagnosis, and only follow-up imaging is performed because it rarely causes clinical symptoms. Conversely, when clinical symptoms occur, they include inflammation, bleeding, obstruction, and malignant transformation [5]; therefore, a biopsy for a definitive diagnosis as well as treatment is needed. Guillou et al. and Makhlouf et al. independently reported that the incidence of malignancy due to HP was 0.7% and 1.8%, respectively [6, 7]. Malignant transformation of the HP needs to fulfill the following histopathologic criteria for diagnosis [6], as occurred in our case:

(1) Tumor found within or close to the heterotopic pancreatic tissue

(2) Direct transition observed between the pancreatic structures and the carcinoma (malignant transformation of the HP must be differentiated from a metastatic deposit or a neoplastic invasion from a neighboring digestive cancer, especially from the stomach, biliary tract, and heterotopic pancreas)

(3) Non-neoplastic pancreatic tissue must comprise at least fully developed acini and ductal structures.

We found 12 other case reports of HP cancer on PubMed records from the year 2000 to 2020, as shown in Table 1 [3, 5, 7–15]. In those reports, the patients’ mean age was 69.7 years (range, 56–86 years). Eight patients were male and 5 were female. As far as the other reports have described, there were 7 cases of type I and one case each of type III and type IV as per the Heinrich classification of HP. All patients presented with clinical symptoms, such as abdominal pain or vomiting because of obstruction caused by the tumor. The mean tumor size was 30.4 mm (range, 12–55 mm). In few cases, serum levels of tumor-specific antigens were not elevated. Almost all patients had preoperative suspicion of malignancy, and only 2 could be confirmed by endoscopic ultrasonography (EUS). No cases of HP adenocarcinoma could be preoperatively diagnosed. Tumor location and the extent of invasion in the pancreas during the surgical procedure produced changes in the surgical plan. In addition, there were 2 cases of gastrectomy, 6 cases of pancreaticoduodenectomy (including SSPPD), and 5 cases of partial duodenectomy. Surgery with lymphadenectomy occurred in 9 cases, and 5 out of 9 cases had metastases to the lymph nodes. Although no standard chemotherapy for malignant HP has been established, 5 among the 13 cases received adjuvant chemotherapy. The mean time of progression-free survival (PFS) was 35 months.

**Table 1:** Summary of the cases of heterotopic pancreas adenocarcinoma in the duodenum found in the literature

Case	Year	Author	Age	Sex	Location	Size (mm)	Biopsy	Preoperative tumor-specific antigen	Diagnostic approach	Lymphadenectomy	Lymph node metastasis	Heinrich classification	Chemotherapy	PFS (months)
1	2007	Tison	72	M	2nd	N.D	Negative	N.D	Ope (PD)	Performed (N.D)	+	N.D	N.D	N.D
2	2007	Kawakami	65	F	2nd	12	Not performed	WNL	Ope (SSPPD)	The regional lymph nodes	N.D	N.D	N.D	19
3	2008	Rosok	59	F	Proximal	50	Negative	N.D	Ope (lap-D)	Not performed	−	N.D	Not performed	36
4	2010	Inoue	75	M	2nd	30	Negative	WNL	Ope (PPPD)	The regional lymph nodes	+	III	Not performed	72
5	2010	Bini	56	M	1st	N.D	Adenocarcinoma	WNL	EUS-FNA → Ope (PD)	The regional lymph nodes	−	I	Performed (N.D)	N.D
6	2011	Stock	79	F	4th	30	Negative	Chromogranin-A, 53 mg/mL	Ope (D)	The regional lymph nodes	+	I	Performed (N.D)	N.D
7	2012	Kinoshita	62	F	1st	34	Negative	CEA, 8.1 U/mL	Ope (PD)	The regional lymph nodes	−	I	Not performed	12
								CA19-9, 66.9 U/mL						
8	2013	Ginori	86	F	1st	30	Not performed	N.D	Ope (subTG)	The regional lymph nodes	−	I	Not performed	N.D
9	2013	Alireza	58	M	1st	27	Not performed	N.D	Ope (D)	Not performed	−	N.D	Performed (XELOX)	18
10	2014	Endo	75	M	2nd	22	Adenocarcinoma	CEA, 55.4 U/mL	EUA-FNA → Ope (SSPPD)	N.D	N.D	I	Not performed	60
								CA19-9, 54.8 U/mL						
11	2015	Fukino	62	M	4th	15	Negative	CA19-9, 500 U/mL	Ope (D)	N.D	N.D	IV	Performed (SP)	N.D
								DUPAN-2, 226 U/mL						
12	2019	Kaneko	81	M	1st	30	Negative	WNL	Ope (DG)	The regional lymph nodes	+	I	Not performed	18
13	2020	Our case	77	M	1st	55	Negative	CA19-9, 1130 U/mL	Ope (SSPPD)	The regional lymph nodes	+	I	Performed (TS1)	9
								DUPAN-2, 5287 U/mL						

*CEA* carcinoembryonic antigen, *CA* carbohydrate antigen, *DUPAN* duke pancreatic monoclonal antigen, *EUS-FNA* endoscopic ultrasonography-guided fine-needle aspiration, *N.D* not dated, *PFS* progression-free survival, *D* (partial) duodenectomy, *DG* distal gastrectomy, *TG* total gastrectomy, *PD* pancreatoduodenectomy, *PPPD* pylorus-preserving pancreatoduodenectomy, *SSPPD* subtotal stomach-preserving pancreatoduodenectomy, *SMT* submucosal tumors, *TS1* tegafur-gimeracil-dihydropyrimidine dehydrogenase, *SP* TS1+cisplatin, *XELOX* capecitabine+oxaliplatin, *WNL* within normal limits

In our case, because of the high levels of serum tumor-specific antigens, we suspected the existence of a malignant tumor. Remarkably, DUPAN-2 being a specific pancreatic tumor antigen, serves as a useful tool for detecting orthotopic pancreatic cancer as well as HP cancer. CT and endoscopy imaging examination initially showed the thickened wall in the first portion of the duodenum. However, several biopsies performed at different times could not reveal the location of the malignancy. Hence, the surgery decision was postponed during the 4 months of follow-up until a precise diagnosis could be achieved. Similar to the previous 12 reports, our case could not be preoperatively diagnosed by biopsy because the tumor existed in the submucosal layer, a difficult site to obtain a sample. Endo et al. [16] suggested the usefulness of preoperative EUS-guided fine-needle aspiration (EUS-FNA) to reveal submucosal malignancies, like HP cancer. Also, cytological examination with EUS revealed it in approximately 50% of the cases [17]. However, when tumor obstruction exists, it makes it difficult to pass through the lesion and perform a precise evaluation. In fact, only a few of the reported cases could be diagnosed using EUS-FNA. We believe that diagnostic surgery is still a good choice to evaluate the tumor location and malignancy and preferable to perform with a lymphadenectomy when considering malignant cases.

The recurrence rate of HP cancer is low. Similar to what has been observed in previous reports, the PFS of the HP adenocarcinoma was longer than that of orthotopic pancreatic cancer: this is probably because the tumor enlargement might cause earlier and more diverse symptoms, thus allowing better control of the disease.

## Conclusions

HP adenocarcinoma is rare and difficult to diagnose due to its submucosal location. EUS is a useful procedure to detect SMT, except for lesions in which the tumor causes obstruction. Therefore, in cases where the primary or malignant lesions are difficult to detect, a careful and short-term follow-up, including blood testing and radiologic imaging, is needed. Additionally, diagnostic surgery should be considered as a choice for evaluating the location of the malignant tissue.

## Data Availability

The datasets supporting the conclusions of this article are included in the article.

## Abbreviations

CA: Carbohydrate antigen
DUPAN: Duke pancreatic monoclonal antigen
EUS: Endoscopic ultrasonography
EUS-FNA: Endoscopic ultrasonography-guided fine-needle aspiration
HP: Heterotopic pancreas
GDA: Gastroduodenal artery
PFS: Progression-free survival
SSPPD: Subtotal stomach-preserving pancreatoduodenectomy
SMT: Submucosal tumors
TS-1: Tegafur-gimeracil-dihydropyrimidine dehydrogenase
